# Identification of HIBCH and MGME1 as Mitochondrial Dynamics‐Related Biomarkers in Alzheimer's Disease Via Integrated Bioinformatics Analysis

**DOI:** 10.1049/syb2.70018

**Published:** 2025-04-26

**Authors:** Hailong Li, Fei Feng, Shoupin Xie, Yanping Ma, Yafeng Wang, Fan Zhang, Hongyan Wu, Shenghui Huang

**Affiliations:** ^1^ Department of General Practice Luohu Clinical College School of Medicine Shantou University Shenzhen China; ^2^ Department of Geriatrics Affiliated Hospital of Gansu University of Traditional Chinese Medicine Lanzhou China; ^3^ Sleep Medicine Ward Shenzhen Kangning Hospital Shenzhen China; ^4^ Department of Neurology Lanzhou First People's Hospital Lanzhou China; ^5^ Department of Geriatrics Luohu District Hospital of Traditional Chinese Medicine Shenzhen China; ^6^ Department of Mental Health and Sleep Center Gansu Provincial Hospital of Traditional Chinese Medicine Lanzhou China

**Keywords:** bioinformatics, biomechanics, brain, data analysis, network analysis

## Abstract

Mitochondrial dynamics (MD) play a crucial role in the genesis of Alzheimer's disease (AD); however, the molecular mechanisms underlying MD dysregulation in AD remain unclear. This study aimed to identify critical molecules of MD that contribute to AD progression using GEO data and bioinformatics approaches. The GSE63061 dataset comparing AD patients with healthy controls was analysed, WGCNA was employed to identify co‐expression modules and differentially expressed genes (DEGs) and LASSO model was developed and verified using the DEGs to screen for potential biomarkers. A PPI network was built to predict upstream miRNAs, which were experimentally validated using luciferase reporter assays. A total of 3518 DEGs were identified (2209 upregulated, 1309 downregulated; |log_2_FC| > 1.5, adjusted *p* < 0.05). WGCNA revealed 160 MD‐related genes. LASSO regression selected HIBCH and MGME1 as novel biomarkers with significant downregulation in AD (fold change > 2, *p* < 0.001). KEGG enrichment analysis highlighted pathways associated with neurodegeneration. Luciferase assays confirmed direct binding of miR‐922 to the 3′UTR of MGME1. HIBCH and MGME1 are promising diagnostic biomarkers for AD with AUC values of 0.73 and 0.74. Mechanistically, miR‐922 was experimentally validated to directly bind MGME1 3′UTR.

## Graphical abstract

HIBCH and MGME1 were found to be considerably downregulated in AD, and miRNA‐922 was found to regulate MGME1.

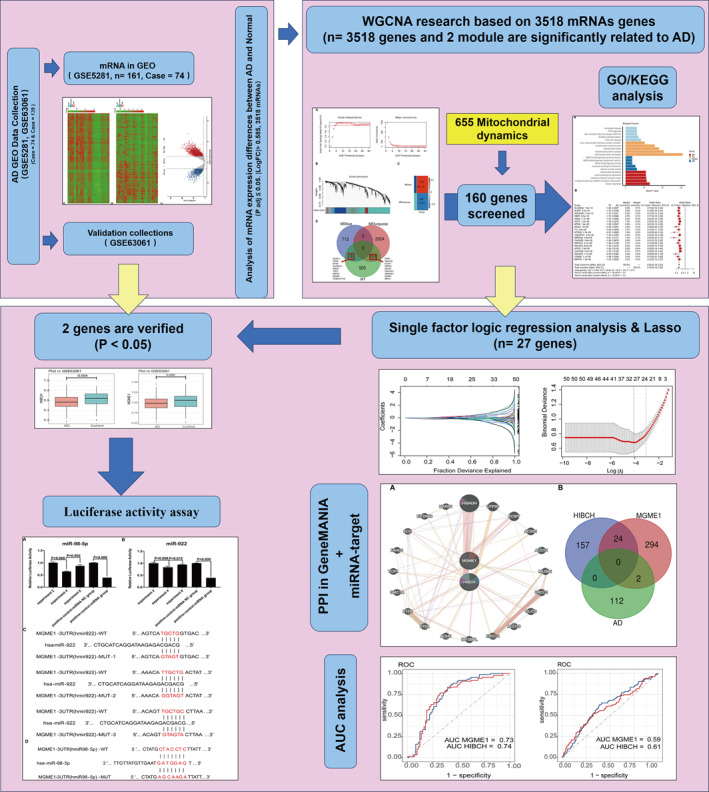

## 1 Introduction

As a progressive neurodegenerative disorder, Alzheimer's disease (AD) characterised by extracellular amyloid‐beta plaques and neurofibrillary tangles has significantly impacted public health worldwide. Epidemiology‐wise, its incidence rate is rising annually due to the exacerbation of population ageing, and it has developed into a significant health issue that has a negative impact on social and economic advancement as well as the quality of life for the aged. Delaying the disease's course and improving patient outcomes depend heavily on early diagnosis [1]. Although newly discovered biomarkers were reported year by year [2], additional evidence, such as Aβ42, p‐tau181, and NfL was obtained and promoted [3, 4]. Currently, there are certain clear drawbacks, such as a lack of early diagnostic sensitivity and specificity in the development and implementation of Alzheimer's disease diagnostic indicators. As biomarker research has advanced in recent years, a number of possible diagnostic markers, including as miRNAs, proteins, metabolites, and other sorts of biological components, have been found. Current biomarkers show limited diagnostic accuracy in early disease stages, highlighting the need for novel molecular signatures. As a result, there is an urgent need to identify new and more accurate diagnostic markers. The fast growth of bioinformatics tools and omics technology has created new prospects for the investigation and development of AD biomarkers or drug sensitivity targets [5, 6, 7]. Bioinformatics technologies can mine possible disease‐related genes and markers by analysing enormous amounts of biological data; for example, graph neural networks [8] and multi‐modal deep learning [9, 10] have been used in the discovery of disease biomarkers. Omics approaches such as genomics, transcriptomics and proteomics may fully explain the mechanism of illness occurrence and potential markers from different levels.

Mitochondrial dysfunction contributes significantly to the aetiology of AD, and its characteristics include disrupted mitochondrial fusion and fission, the generation of reactive oxygen species (ROS), as well as mitochondrial transport and reduction of the activity of mitochondrial complexes [11]. Recent studies highlight the role of mitochondrial DNA (mtDNA) maintenance enzymes in neurodegeneration. Alterations in mitochondrial dynamics have been seen in both AD [12], as well as in Parkinson's disease [13]. Potential novel approaches for diagnosing and treating mitochondrial abnormalities in AD are being studied [14, 15]. Mitochondrial dynamics imbalance has been implicated in synaptic dysfunction and neuronal death through impaired energy metabolism and increased oxidative stress. In the future, maintaining mitochondrial homoeostasis appears to be an appealing therapeutic target [15, 16]. The aetiology of early AD, strategies for maintaining mitochondrial homoeostasis and mitochondrial targeting approaches are all impacted by mitochondrial homoeostasis disorders [16].

MicroRNA (miRNA) is an essential regulatory molecule that has a function in the aetiology of Alzheimer's disease. In recent years, miRNA biomarkers have received a lot of attention in the field of AD research. In short, an in‐depth study of the epidemiology of AD, overcoming limitations in diagnostic marker research, and using bioinformatics methods and omics technologies to develop effective markers, including miRNA markers, are critical for improving the diagnostic level of AD and patient prognosis. The goal of this work was to use bioinformatics to identify newly found AD biomarkers related to mitochondrial dynamics from the GEO database.

## 2 Results

### 2.1 Exploration of Differential Genes Between AD and Healthy Populations

**FIGURE 1:**
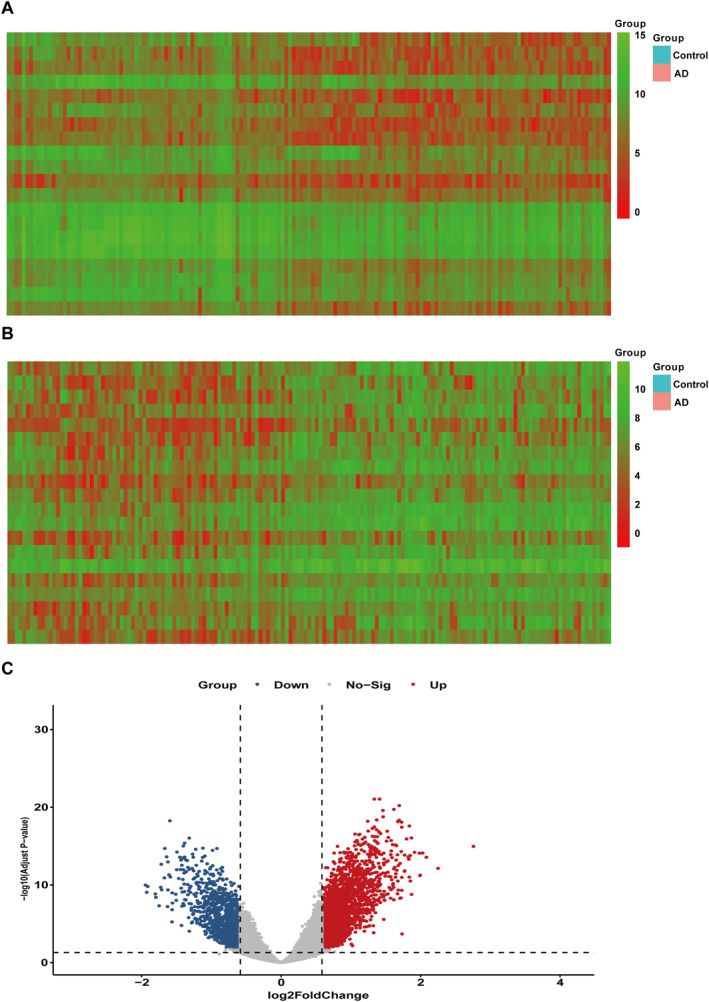
The volcano plot of significantly different mRNA genes and significant difference mRNA heatmap. (A) The volcano plot of significantly different mRNA genes, including 2209 upregulated genes (B) 1309 downregulated genes, as shown in Figure 1B (|logFC| > 0.585, *p* adj < 0.05). (C) All differently expressed genes were also illustrated as heatmap.

To explore the differences in gene expression between AD patients and healthy populations, the study was based on GEO data from 161 training sets, including 74 AD patients. A total of 3518 significantly differentially expressed genes were identified, of which 2209 genes were upregulated and 1309 genes were downregulated (|logFC| > 0.585), *p* adj < 0.05, as illustrated in Figure 1A–C.

### 2.2 WGCNA Screening the Mitochondrial Dynamic‐Related Genes in AD

**FIGURE 2:**
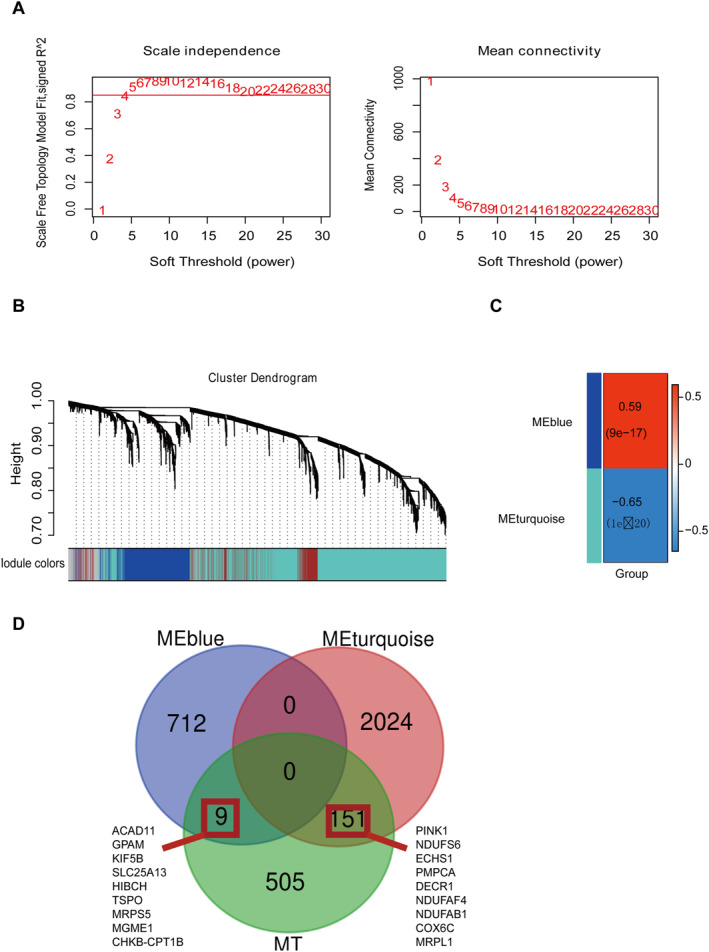
WGCNA analysis and VENN analysis of WGCNA module genes and mitochondrial dynamics genes. (A) A total of 3518 mRNA transcripts across 158 samples were included in subsequent analyses. Weighted gene co‐expression network analysis (WGCNA) was performed with a soft‐thresholding power of 5. (B) This identified four co‐expression modules containing 240, 2,175, 721, and 382 genes, respectively. (C) Modules MEblue and Menturquoise showed significant association with AD pathology, exhibiting 2896 differentially co‐expressed genes. (D) Venn diagram overlap analysis identifies 160 mitochondria dynamics‐associated hub genes in AD, including key players such as HIBCH and MGME1.

This study investigated the expression matrix of 3518 significantly differentially correlated gene sets based on a training set of 161 samples to discover the substantial relationship between differentially expressed gene networks and AD patients, and WGCNA was explored as well. Firstly, the top 75% genes with absolute median deviation were excluded from the screening, with MAD greater than 0.01. Three outlier samples were excluded for WGCNA analysis, including “GSM119666,” “GSM119676” and “GSM238941.” The study ultimately included 3518 mRNA gene expression matrices from 158 samples for subsequent analysis. Afterwards, *R*‐square = 0.85 was set to determine the soft threshold Power = 5 (Figure 2A) for co‐expression network analysis. The WGCNA calculation results indicated four effective clustering modules (WGCNA/Network Rating. csv). (Figure 2B). Among them, there are 240, 2175, 721, and 382 genes, respectively. Of these, there is a significant correlation between genes in the two modules of MEblue and Menturquoise and AD, collectively containing 2896 genes (*p* < 0.01, |Coef| > 0.3, Figure 2C). Subsequently, the study used 665 mitochondrial dynamic related genes and the aforementioned 2896 co‐expression sets for VENNY analysis, and the results indicated 160 WGCNA clustering genes related to mitochondrial dynamic, as illustrated in Figure 2D.

### 2.3 GO/KEGG of Differentially Expressed Genes

**FIGURE 3:**
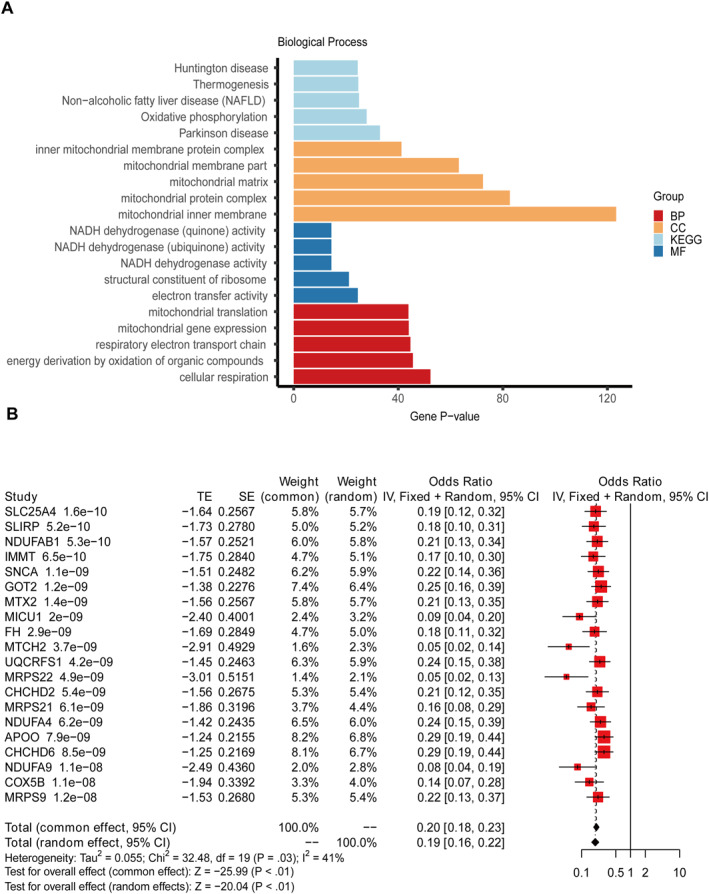
GO/KEGG analysis and univariate logistic regression analysis of differentially expressed genes. (A) The KEGG and GO analysis results indicated 385 significantly enriched pathways (*p* < 0.05, Count > 2). (B) The first 20 genes of 160 genes conducted single factor logistic regression were illustrated as the forest map.

Based on the above 160 gene results, a pathway enrichment study was conducted on KEGG and GO, and the results indicated 385 significantly enriched pathways (*p* adj < 0.05, Count > 2, Figure 3A). This mainly includes Huntington disease, Thermogenesis, Nonalcoholic fatty liver disease (NAFLD), etc.

### 2.4 Single Factor Logistic Regression Analysis

Based on the 160 candidate genes mentioned above, the study used univariate logistic regression to explore the relationship between AD and healthy individuals. A threshold *p* value < 0.01 was set, and 160 genes were ultimately included for further research. Univariate logistic regression analysis/univariate results. csv). Display the forest map for the first 20 terms, as illustrated in Figure 3B. Subsequently, 160 genes significantly correlated with single factors were included in subsequent studies.

### 2.5 Lasso Model Construction and Validation

**FIGURE 4:**
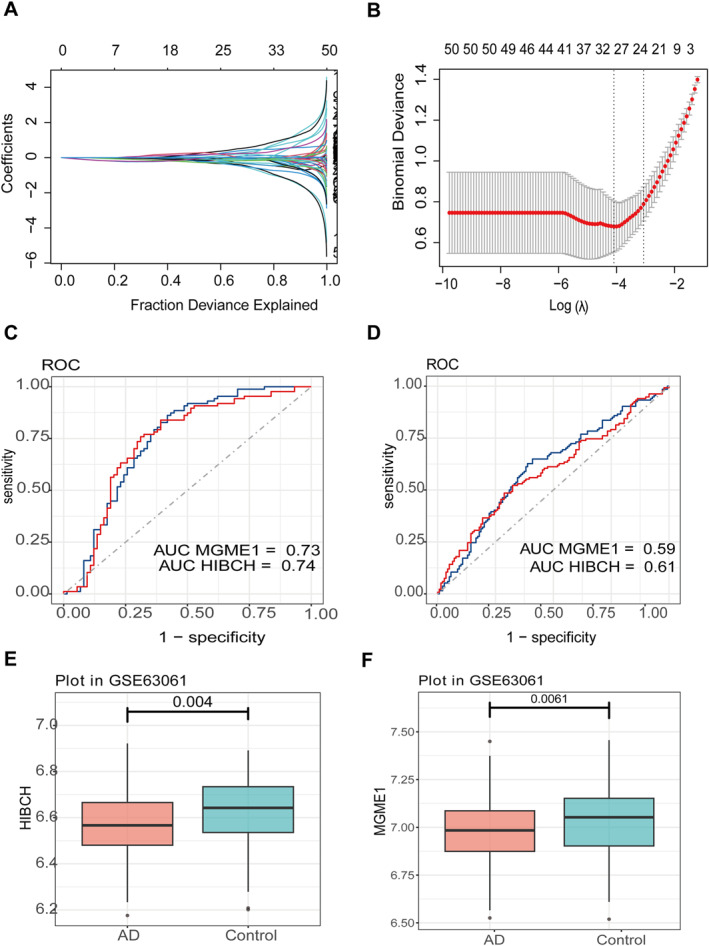
Diagnostic performance of HIBCH/MGME1 in AD. (A, B) Lasso regression identified 27 candidate genes. (C, D) ROC curves validated HIBCH/MGME1 as robust biomarkers (AUC = 0.73–0.74). (E, F) HIBCH and MGME1 expression is significantly downregulated in AD tissues.

Based on the 160 single factors, AD was significantly correlated with validated mitochondrial dynamic genes with significant differences and consistent directions. Lasso was used for feature screening to determine 27 features, as illustrated in Figure 4A,B. Based on 27 screened genes, the expression values of two genes HIBCH and MGME1 were identified in two sets (validation set and training set), with the highest HIBCH value of 0.61 in the validation set as illustrated in Figure 4C,D. HIBCH and MGME1 showed consistent and significant downregulation in gene expression in the validation set GSE63061, and we included them in subsequent studies, as illustrated in Figure 4E,F.

### 2.6 PPI Construction of Key Genes

**FIGURE 5:**
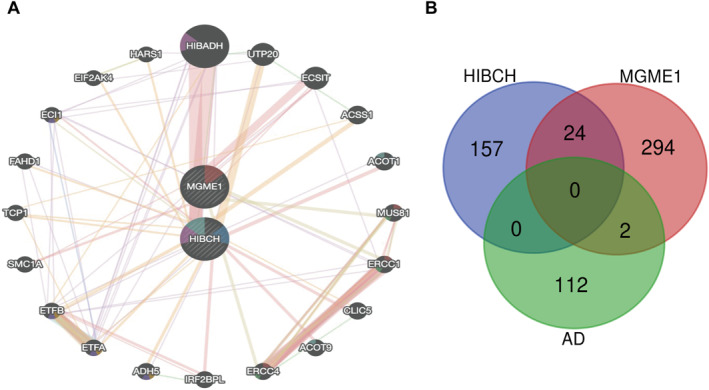
Integration of protein‐protein interactions (PPI) and miRNA regulatory networks. (A) PPI network analysis (GeneMANIA) identified key interactors of diagnostic genes, highlighting functional associations (co‐localization, shared domains, and pathways). (B) miRNA‐target prediction identified hsa‐miR‐922 and hsa‐miR‐98 as upstream regulators of MGME1 through cross‐database validation (mirWalk).

Based on the above two genes (HIBCH and MGME1), a PPI analysis was conducted using the GeneMANIA database to predict the correlation between diagnostic genes and their 20 interacting genes, including colocalisation, shared protein domains, co‐expression, prediction and pathways. The research results indicate that there are multiple interactions between the two genes validated in the external validation set, such as physical interactions and co‐expression, including HIBADH, UTP20, ACSS1 and other genes, as illustrated in Figure 5A.

### 2.7 Prediction of Upstream miRNAs of Key Genes

To explore whether there are targeted miRNAs in the regions of two genes (HIBCH and MGME1), the study used the above method to explore 1023 candidate miRNAs that are significantly correlated with two validation genes in the mirWalk database, and identified the intersection of two candidate genes and 125 miRNAs of the disease. Two miRNAs were selected for subsequent studies, including: has‐miR‐922, has‐miR‐98, as illustrated in Figure 5B.

### 2.8 Transcription Factor Prediction Analysis

Based on the transcription factor prediction provided by iRegulon in Cytoscape, set Enrichment score threshold = 3.0/2.0, ROC threshold for AUC calculation = 0.03, Rank threshold = 5000, No significant interaction TF factor between the two genes was found in the predicted results.

### 2.9 Small Molecule Drug Prediction and Molecular Docking Analysis

**FIGURE 6:**
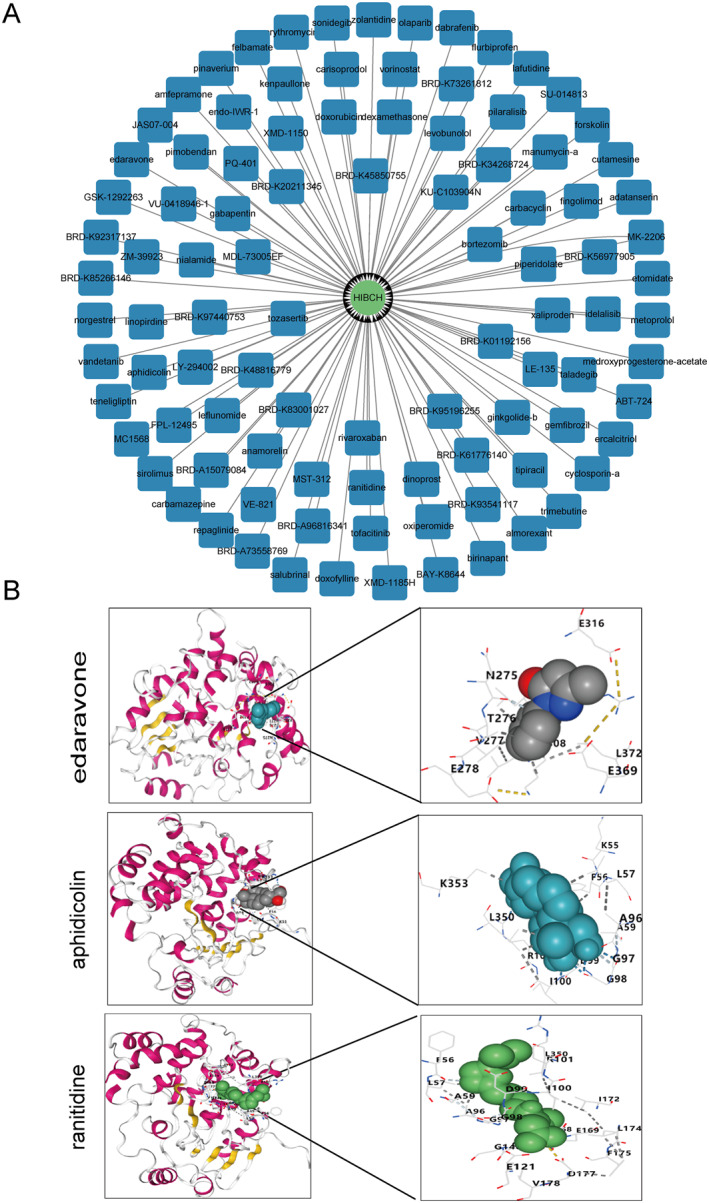
Small‐molecule drug prediction for candidate genes. (A) Signature Search identified 100 drug chemical small molecules related to expression regulation of HIBCH (Z Score > 1, *p* < 0.1). (B) Edaravone, aphidicolin, and ranitidine as top regulators of HIBCH (Z Score > 1, *p* < 0.1).

To explore drug chemical small molecules related to candidate genes, two model genes were predicted using Signature Search. Only the HIBCH gene was enriched with 100 drug chemical small molecules related to expression regulation (*Z* Score > 1, *p* < 0.1), as shown in Figure 6A, with the most significant being edaravone, aphidicolin and ranitidine, as shown in Figure 6B, and no chemical small molecules of MGME1 could be predicted. Molecular docking showed that the vina score of edaravone, aphidicolin and ranitidine with HIBCH were mostly less than −5 kcal/mol, reflecting the binding capacity (Tables 1, 2, 3).

**TABLE 1:** The parameters of molecular dynamics analysis of the vina score of edaravone, with HIBCH.

CurPocket ID	Vina score	Cavity volume (A3)	Centre (*x*, *y*, *z*)	Docking size (*x*, *y*, *z*)
C4	−6.2	293	34, −5, 35	18, 18, 18
C1	−5.9	633	43, 0, 11	18, 18, 18
C5	−5.7	224	29, −16, 17	18, 18, 18
C2	−5.3	401	47, 2, 27	18, 18, 18
C3	−4.9	360	31, 8, 26	18, 18, 18

**TABLE 2:** The parameters of molecular dynamics analysis for the vina score for aphidicolin with HIBCH.

CurPocket ID	Vina score	Cavity volume (A3)	Centre (*x*, *y*, *z*)	Docking size (*x*, *y*, *z*)
C1	−6.9	633	43, 0, 11	21, 21, 21
C5	−6.7	224	229, −16, 17	21, 21, 21
C4	−6.2	293	34, −5, 35	21, 21, 21
C2	−6.1	401	47, 2, 27	21, 21, 21
C3	−6.0	360	31, 8, 26	21, 21, 21

**TABLE 3:** The parameters of molecular dynamics analysis for the vina score for ranitidine with HIBCH.

CurPocket ID	Vina score	Cavity volume (A3)	Centre (*x*, *y*, *z*)	Docking size (*x*, *y*, *z*)
C4	−6.2	633	43, 0, 11	21, 21, 21
C1	−5.9	224	29, −16, 17	21, 21, 21
C5	−5.7	293	34, −5, 35	21, 21, 21
C2	−5.3	401	47, 2, 27	21, 21, 21
C3	−4.9	360	31, 8, 26	18, 18, 18

### 2.10 Co‐Transfection of miNA and MGME1 3‐'UTR Report Plasmid Vector

293T cells were prepared, digested and collected by trypsin, and then made into a cell suspension and added to 24‐well plates. The number of cells per well amounts to 5 × 10^4^. When the cell density reached to 90%–95%, the cells were transfected. The experiment was divided into six groups: MAPK1 3′‐UTR‐NC + mir‐NC group, MGME11 3′‐UTR‐NC + mir‐922/mir‐98‐5p group, MGME11 3′‐UTR ‐WT + mir‐NC group, MGME11 3′‐UTR‐WT + mir‐922/mir‐98‐5p group, MGME11 3 ‐' UTR‐MUT + mir‐NC group and MGME11 3′‐UTR‐MUT + mir‐922/mir‐98‐5p group. The amount of plasmid was 0.5 μg/well, and the amount of liposome was 1.5 μL/well. In each group, 3 multiple holes were set, and the experiment was repeated 3 times.

### 2.11 miR‐922 Directly Regulates MGME1 by Luciferase Activity Assay

**FIGURE 7:**
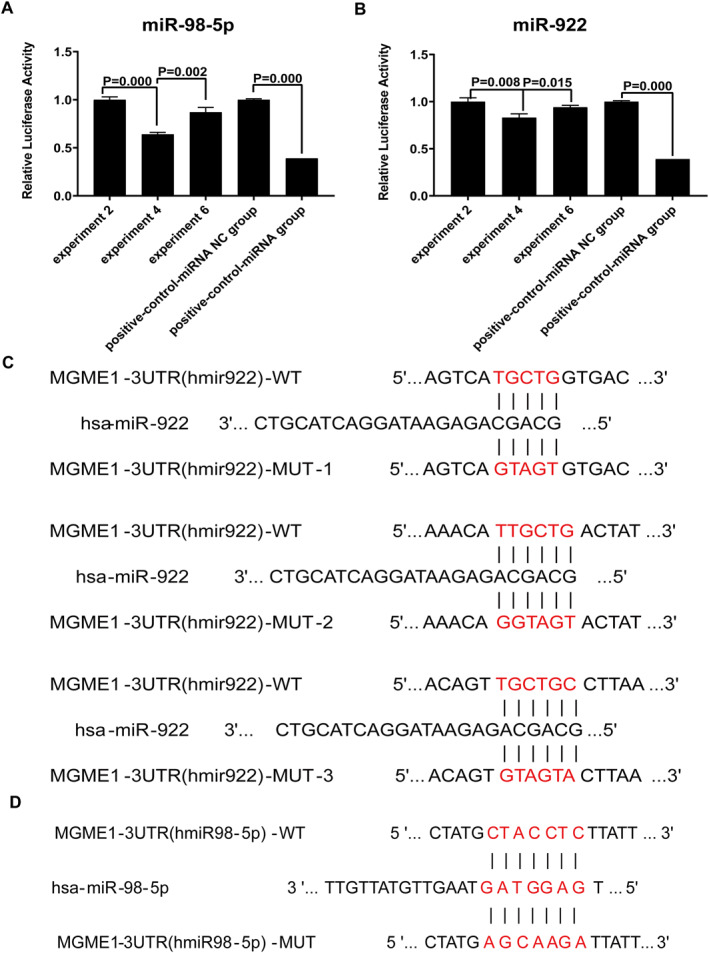
Luciferase reporter assay validation of miRNA‐MGME1 binding. (A, C) miR‐922 directly binds to the MGME1 3′UTR: Wild‐type (WT) versus mutant (MUT) plasmids: Luciferase activity decreased by 42% (*p* = 0.003). Negative control (NC) group validated system integrity. (B, D) miR‐98‐5p shows no specific binding: Activity reduction < 20% versus NC (*p* > 0.05), confirming non‐specific effects. miR‐922, but not miR‐98‐5p, post‐transcriptionally suppresses MGME1 expression via direct 3′UTR interaction.

In order to determine whether miR‐922 and miR‐98 directly regulates MGME1 further, we constructed wild‐type and mutant plasmids of MGME1 and carried out double luciferase experiments. The results showed that compared with 3′‐UTR‐WT + miR‐NC, the fluorescence value of MGME1 3′‐UTR‐WT + miR‐922 decreased significantly (*p* < 0.01); compared with MGME1 3′‐UTR‐Mut + miR‐NC, the fluorescence value of MGME1 3′‐UTR‐Mut + miR‐922 group did not change significantly (*p* > 0.05). The results showed that compared with 3′‐UTR‐WT + miR‐NC, the fluorescence value of MGME1 3′‐UTR‐WT + miR‐98‐5p decreased but the change was not higher than 20% (*p* < 0.01); compared with MGME1 3′‐UTR‐Mut + miR‐NC, the fluorescence value of MGME1 3′‐UTR‐Mut + miR‐922 group did not change significantly (*p* > 0.05). The results showed that miR‐922 could bind to the 3 ‘UTR of MGME1 and inhibit its expression. Overall, miR‐922 could bind to the 3 ‘UTR of MGME1, as shown in Figure 7A, C. However, in the same detection system, miR‐98‐5p cannot bind to the 3 ‘UTR of MGME1, as shown in Figure 7B,D and Table 4.

**TABLE 4:** Experiment treatment of different groups.

Group	Experiment treatment
Experiment 1	Luc‐MGME1‐3′ UTR (hsa‐miRNA)‐WT‐3′UTR‐NC + hsa‐miR‐98‐5p‐NC
Experiment 2	Luc‐MGME1‐3′ UTR (hsa‐miRNA)‐WT‐3′UTR‐NC + hsa‐miRNA
Experiment 3	Luc‐MGME1‐3′ UTR (hsa‐miRNA)‐WT‐3′UTR + hsa‐miRNA‐NC
Experiment 4	Luc‐MGME1‐3′ UTR (hsa‐miRNA)‐WT‐3′UTR + hsa‐miRNA
Experiment 5	Luc‐MGME1‐3′ UTR (hsa‐miRNA)‐MUT‐3′UTR Mut + hsa‐miRNA‐NC
Experiment 6	Luc‐MGME1‐3′ UTR (hsa‐miRNA)‐MUT‐3′UTR Mut + hsa‐miRNA
Positive control miRNA NC group	Positive control 3′ UTR + miRNA‐NC
Positive control miRNA group	Positive control 3′ UTR + Positive control miRNA

*Note:* miRNA‐NC: microRNA empty vector, used as a negative control for the target microRNA plasmid, miRNA: MicroRNA vector plasmid, expressing the target microRNA (hsa‐mir‐98‐5p or hsa‐mir‐922), 3′ UTR‐NC: 3′ UTR empty plasmid, used as a negative control for the target gene 3′ UTR plasmid, 3′ UTR: Target gene 3′ UTR plasmid (MGME1), 3′ UTR‐MU: Target gene 3′ UTR mutant plasmid (MGME1), Positive control 3′ UTR: TRAF6 gene, Positive control miRNA 3′ UTR plasmid, Hsa‐mir‐146b vector plasmid.

## 3 Discussion

Through screening and validation in the test and validation sets, it was discovered that mitochondrial genome maintenance exonuclease 1 (MGME1, also known as DDK1) was significantly downregulated in AD patients. This study employed bioinformatics methods to study AD clinical samples in the GEO database. It was discovered that mir‐922 can target and regulate MGME1 by examining the miRNA target library and the miRNA disease database of AD in order to identify the intersecting genes. Thus, we hypothesise that the high expression of mir‐922 may be controlling the downregulation of MGME1 expression in AD patients.

The MGME1 gene, located at 20p11.23, encodes RecB type 5 ′‐3′ nuclease, a single‐stranded DNA (ssDNA) exonuclease that is involved in mitochondrial genome maintenance. MGME1 is capable of cutting many DNA or DNA‐RNA chimaeric oligonucleotides with free nucleic acid ends. This enzyme can also cleave single‐stranded DNA (ssDNA). It may contribute to mtDNA repair by processing DNA strands during long patch base excision repair or displaced DNA, such as Okazaki fragments, during RNA‐induced DNA synthesis on lagging strans. Mammalian mitochondrial DNA replication is a critical process that must be performed with high fidelity and under several restrictions to ensure optimal mitochondrial function. Previous research studies have indicated that MGME1 and additional enzymatic DNA repair processes are essential for the replication and maintenance of mtDNA. Empirical research has indicated the pivotal role that MGME1 and additional enzymatic DNA repair mechanisms perform in the replication and maintenance of mtDNA. Although single and multiple mtDNA deletions can be caused by homozygous ablation of the MGME1 gene, issues with mtDNA replication and maintenance can arise from MGME1 mutations. The repair proteins and mtDNA replicons may have defects that lead to diseases related to mtDNA instability. Different clinical diseases associated with one or more mtDNA deletions are usually caused by allelic mutations in these genes [17].

The replication of mitochondrial genomes depends on MGME1‐mediated mtDNA repair, which may also be a prerequisite for intramolecular recombination of mtDNA molecules. Diseases brought on by pathogenic variations of nuclear genes preserved by mtDNA are linked to MGME1. Deficiencies or mutations in MGME1 limit the synthesis of mtDNA, which results in mitochondrial DNA maintenance deficiencies [17]. A protracted 7S DNA accumulation occurs once MGME1 is lost due to insufficient 5′ end processing. At the mtDNA terminal, homologous recombination repair may include MGME1. Mitochondrial replicase PolgA can interact with MGME1 to increase its exonuclease activity. The nucleic acid penetrant activity of restriction endonucleases causes linear mtDNA to be eliminated after a few hours in the cell model of mtDNA double‐strand breaks. Addition of *p* results in the inactivation of the mitochondrial 5′‐3′ monoribozyme MGME1. Significant obstacles to the breakdown of mitochondrial DNA are presented by the D274 A mutation, which inhibits the 3′‐5′ mononuclear protease activity of mitochondrial DNA polymerase POLG or depletes mitochondrial DNA helicase TWNK [18]. MGME1 is involved in the elimination of linear mtDNA; alternatively, a different possible mitochondrial nuclease could generate single‐stranded mtDNA, which would then serve as MGME1's substrate [19]. Moreover, MGME1 knockout mice accumulate long line mtDNA fragments and multiple deletions, which show a highly tissue‐specific replication arrest effect on mtDNA [20].

According to clinical study, mutations in the MGME1 gene may cause mitochondrial DNA depletion syndrome type 11 (MTDPS11). For instance, a patient, at the age of 11, with the MGME1 mutation, which presented as MTDPS11, started to have learning difficulties, frequent stumbles and slowly moving lower eyelids. Physical examination findings included elbow hyperextension, flat feet, mild scoliosis, persistent progressive external ophthalmoplegia with upper eyelid ptosis and global hypotonia [21]. Muscle biopsies from patients, blood and urine samples from family I, and cells downregulated by MGME1 siRNA all revealed a relative increase in 7S DNA levels and a loss of mtDNA. As such, MGME1 mutations could be a contributing factor to mtDNA‐related diseases [22]. Although nuclear gene mutations or deletions were formerly thought to exclusively cause inherited problems, it has progressively come to light that they may also be responsible for neurodegenerative diseases. MGME1 was identified among 68 signature genes distinguishing Alzheimer's disease (AD) from Parkinson's disease (PD) and healthy controls [23]. Mechanistically, oxidative stress may exacerbate ageing by disrupting nuclear‐encoded mitochondrial DNA (mtDNA) replication machinery—including POLG, POLG2, TWNK, TFAM, RNase H1 and critically MGME1—thereby promoting age‐related pathologies through mtDNA instability [24]. This is substantiated by evidence linking MGME1 mutations to pathological accumulations of mtDNA deletions, which amplify susceptibility to infection‐triggered chronic inflammation—a hallmark of neurodegenerative progression [25]. Notably, MGME1 silencing impairs mtDNA metabolism and suppresses NLRP3 inflammasome activation [26], while simultaneously reducing cytosolic oxidised mtDNA (Ox‐mtDNA) leakage. Furthermore, MGME1 inhibition significantly decreases total mtDNA content in LPS‐primed cells, suggesting its dual role in modulating both mtDNA integrity and inflammatory cascades. In summary, MGME1 deficiency may impair mtDNA repair, leading to mitochondrial genome instability and bioenergetic failure. These findings collectively nominate MGME1 as a promising AD biomarker, with therapeutic targeting of MGME1 potentially mitigating pathological mtDNA‐mediated neuroinflammation. The 3‐hydroxyisobutyryl‐coenzyme A (CoA) hydrolase (HIBCH) enzyme, which is encoded by HIBCH, is involved in significant stages of valine degradation. Neurological symptoms resulting from uncommon metabolic dysfunctions are caused by HIBCH deficit (HIBCHD) [27]. An uncommon condition of the mitochondrial valine metabolism known as 3‐Hydroxyisobutyryl‐CoA hydrolase (HIBCH) deficiency can cause organic aciduria, motor delay, hypotonia, ataxia, dystonia, seizures, poor eating and developmental regression or delay [4]. Both HIBCH deficiency and Leigh/Leigh‐like illness are caused by mutations in the 3‐Hydroxyisobutyryl‐CoA hydrolase (HIBCH) gene [4]. Leigh‐like disease and HIBCH deficiency can be caused by mutations in the 3‐Hydroxyisobutyryl‐CoA hydrolase (HIBCH) gene [4]. Neuroimaging results show abnormalities in signals in the cerebral peduncles and globus pallidi, which are located in the deep grey matter [4]. The findings of neuroimaging reveal anomalies in signals within the deep grey matter, specifically in the cerebral peduncles and globus pallidi [28, 29]. It was discovered that HIBCH is one of the proteins that is overexpressed in malignancies, such as ovarian tumours, and that is expressed differentially in mitochondria [30], Blocking 3‐hydroxyisobutyryl‐CoA hydrolase (HIBCH) to stop the breakdown of valine resulted in decreased intracellular succinate, a decrease in the development of cancerous prostate cells, and impaired cellular respiration [31]. This infantile neurological disorder is related to abnormalities on magnetic resonance imaging (MRI), brain atrophy, hypotonia and developmental delay [32, 33]. HIBCH was reported upregulated in male‐specific DEGs between AD and controls [34]. HIBCH was reported upregulated in HEK293/tau cells with stable overexpressing human wild‐type full‐length tau, and also was one target of Chuanxiong Rhizoma [35], in addition, HIBCH was reported upregulated in the temporal cortex of the AD patients [35]. HIBCH was one of the proteins co‐immunoprecipitating with glucosamine 6 Phosphate Isomerase 2 (GNPDA2), which is involved in the amino‐acid metabolic process, and the presence of mutated Tau‐P301 L in GNPDA2‐overexpressing NECs caused a decrease in proliferative capacity while disrupting protein processing [36]. Therefore, HIBCH was an AD biomarker involved in Tau metabolism. According to bioinformatics result, this study found that miR‐922 could target MGME1 by binding its3′ UTR region, but mir‐98 cannot target MGME1. Therefore, miR‐922/MGME1 axis may influence the function of mitochondrial, and thereby, participate in the pathophysiology of AD. Previous reports showed that miR‐922 was upregulated in AD [37]. The pathophysiology of AD is believed to involve miR‐922, an upstream molecule of MGME1. A higher level of miR‐922 expression in AD suppresses ubiquitin carboxy‐terminal hydrolase L1 in vitro via phosphorylating Tau contribute to Alzheimer's disease aetiology [38]. Despite data suggesting that miR‐98‐5p may contribute to AD by upregulating Nrf2 target genes and boosting α 7‐nAChR, it also alleviated neuroinflammation by blocking the NF‐κB pathway. Nevertheless, the luciferase experiment verified the targeting of miR‐922 alone, not miR‐98‐5p targeting MGME1. In conclusion, this study discovered that HIBCH and MGME1 are new biomarkers of AD, and miR‐922/MGME1 axis may contribute to the pathophysiology of AD, could act as the diagnostic and treatment target for AD.

Impaired mitochondrial function and dynamics exacerbated AD pathology, which was accompanied by dysregulated expression of mitochondrial fission proteins Drp1, Fis1, MFN1/2, and OPA1 in early AD [39]. Aβ pathology significantly impacts human astrocytes, leading to elevated levels of phosphorylated DRP‐1 [40]. Drp1 interacts with Aβ and phosphorylated tau, causing excessive mitochondrial fragmentation. This leads to synaptic dysfunction, neuronal injury and cognitive decline. DRP‐1 is now a therapeutic target for AD [41]. The wild‐type human full‐length tau and non‐acetylated tau mutants promoted mitochondrial fusion by raising the number of mitochondrial fusion proteins. Conversely, acetylated tau mutants with K274/K281 mutations induced mitochondrial fission via decreasing mitochondrial fusion proteins, increasing mitochondrial dysfunction and death [42]. Emerging evidences suggested that mitochondrial function and dynamics have a role in the development of Alzheimer's disease, which is caused by Aβ and phosphorylated tau. The mitochondrial fusion protein has been identified as a potential target for treating AD. Using bioinformation approaches, we discovered HIBCH and MGME1 as novel biomarkers to identify the underlying processes of mitochondrial dynamics that cause impaired mitochondrial function in Alzheimer's disease, and HIBCH was linked to Tau metabolism. Compared to well‐known mitochondrial genes, our study found that HIBCH and MGME1 were most strongly associated with AD.

Although our current study utilised established bioinformatics pipelines (e.g., WGCNA, LASSO regression) to identify mitochondrial dynamics‐related genes such as HIBCH and MGME1, emerging computational frameworks like transformer‐based models holding significant promise for advancing this field [43]. Transformer architectures can be utilised to more precisely prioritise mitochondrial dynamics genes while also evaluating multi‐omics datasets to find nonlinear connections between mitochondrial genes and various disease, such as, AD pathogenesis. These algorithms can also combine several data sources to improve biomarker discovery. Future research could employ these models to validate findings, identify novel mitochondrial dynamics regulators, and refine treatment targets. This collaboration with computational tools will help bridge the gap between mitochondrial biology and AI‐powered precision medicine in neurodegenerative illnesses.

In conclusion, HIBCH and MGME1, two mitochondrial dynamics‐related genes, were identified as novel diagnostic biomarkers for AD, with miR‐922 directly regulating MGME1. The miR‐922/MGME1 axis represents a potential therapeutic target for restoring mitochondrial homoeostasis. Our findings extend previous reports of mitochondrial dysfunction in AD by identifying specific DNA repair deficits, and this finding provides mechanistic insights into mitochondrial dysfunction in AD and highlight potential targets for early diagnosis. The clinical relevance should be validated in larger longitudinal cohorts with CSF biomarker confirmation, such as liquid biopsies for diagnosis and explore therapeutic modulation of miR‐922.

Although our study identified HIBCH and MGME1 as promising biomarkers for AD, several limitations warrant further investigation. First, relying on a single GEO dataset presents the possibility of dataset bias; multi‐centre validation with varied cohorts is required to demonstrate generalisability. In vitro luciferase tests verified the miR‐922 targeting MGME1 regulatory relationship; however, in vivo functional investigations, such as CRISPR‐based knockdown in AD animal models or patient‐derived neurons, are needed to show causality and therapeutic significance. Furthermore, although the diagnostic performance of HIBCH and MGME1 aligns closely with established AD biomarkers such as Aβ42, these mitochondrial dynamics‐related markers may serve as complementary tools to enhance early detection specificity. To strengthen clinical relevance, future works will prioritise validating HIBCH/MGME1 protein levels in CSF and blood samples via ELISA to assess their viability as minimally invasive biomarkers. These steps will clarify their utility in multi‐marker panels and accelerate translation into clinical workflows. In addition.

## 4 Materials and Methods

### 4.1 Data Acquisition and Filtering

Two datasets of Alzheimer's disease (AD) were downloaded from GEO data, of which GSE5281 included 161 samples, including 74 AD samples. The GSE63061 collection consists of 388 samples, including 139 AD samples. The study mainly included a gene expression matrix (gene chip data) and accompanying population categorisation data. The downloaded GEO gene expression chip data were subjected to log2 transformation standardisation, and then the data were normalised using the normalise between Arrays function provided in the R language limma 3.9.19 package. Afterwards, the genes in the above dataset were annotated using the chip annotation files of GPL96 and GPL570 provided by GEO. At the same time, the identical probes were merged based on the mean expression value. To reduce the influence of missing values on the results, probes with 20% missing from the entire sample were deleted. The GSE5281 collection had a total of 22,880 probes for later analysis, whereas the GSE63061 collection comprised 16,032 probes. To address the issue of sample distribution, the study employed the GSE5281 dataset as the training set and GSE63061 as the validation set. This collection includes 139 AD and 134 control (healthy) labelled samples for validation, leaving out 115 additional samples. In addition, the study also obtained the GeneCards database (http://www.cancertelsys.org/telnet/) to retrieve 665 genes related to mitochondrial dynamics for subsequent research (score > 10).

### 4.2 Exploration of Differential Genes Between AD and Healthy Populations

The R language limna package (v3.44.3, https://bioinf.wehi.edu.au/limma/) was used to perform mRNA expression differential analysis on AD versus normal during GSE5281 training and create a differential expression volcano map in order to find significantly differentially expressed mRNA in AD patients. Ultimately, the genes selected for further analysis were those with Log|FC| > 0.585 and *p* adj < 0.05.

### 4.3 WGCNA Screening for AD Related Studies

Based on the gene sets with significant expression differences mentioned above, the weighted gene co‐expression network analysis (WGCNA) was conducted to explore the co‐expression status of gene sets with significant differences between AD and control populations. Based on the WGCNA exploration results, the differential gene module network for AD were analysed, which is a systems biology method used to describe gene association patterns between different samples. Thereby, the WGCNA method could be used to identify gene sets with highly synergistic changes and to identify candidate biomarker genes or therapeutic targets based on the interconnectedness of gene sets and the association between gene sets and phenotypes. To increase the reliability of network construction, the study first used the “WGCNA” package (v 1.71) for gene screening and quality control of gene expression values, mainly based on the top 75% of the median absolute deviation genes with at least a MAD greater than 0.01. Afterwards, the hclust clustering of the gene row sample population was performed using the sample tree function within the package. Threshold Z. k was set to −2.5 to remove outlier samples (“GSM119666, GSM119676, and GSM238941”) to prevent subsequent soft threshold screening differences. Based on the above quality control processes, the study first constructed a co‐expression network (weighted gene network) of the differentially expressed mRNA genes between the AD and control populations, where points represent genes and edges represent gene expression correlations. The weighted dark operation (soft threshold, power) uses the pickSoft threshold function of the *R* language “WGCNA” package to determine the appropriate power (*R*‐square > 0.80) and construct the network (maxBlockSize = 5000). The calculation method for the edge properties of undirected networks is given by the following equation:

(1) $${\text{Abs}\left(\text{Cor}\left(\text{genex},\text{geney}\right)\right)}^{\text{power}}$$

The calculation method for edge properties of directed networks is given by the following equation:

(2) $${\left(1+\frac{\text{Cor}\left(\text{genex},\text{geney}\right)}{2}\right)}^{\text{power}}$$

The edge attribute calculation method for sign hybrid is given by the following equation:

(3) $${\;\text{Cor}\left(\text{genex},\text{geney}\right)}^{\text{power}}\;\text{if}\;\text{Cor}>0\;\text{else}\;0$$

Finally, the plotDendro and Colours were used to draw the hierarchical clustering of gene modules. After identifying all differentially expressed gene module networks in the disease, this study also conducted correlation tests between case‐control traits and gene module networks. The study primarily explored gene modules strongly associated with AD using the correlation technique supplied by WGCNA. In parallel, the intersection genes associated to mitochondrial dynamics was extracted in order to identify genes that were differently expressed in relation to disease‐related mitochondrial dynamics (*p* < 0.01, |Coeff| > 0.3).

### 4.4 GO/KEGG of Differentially Expressed Genes

Based on the WGCNA method, the mitochondrial dynamic‐related genes with significant differences were identified through the R language clusterProfiler package (v 3.16.1; https://guangchuangyu.github.io/software/clusterProfiler/). Using the following database sources, Home Sapiens Entrez gene IDs were utilised to convert gene IDs and carry out pathway and process enrichment analysis on the gene list: In order to detect enrichment items with substantial changes (*p* < 0.05), the GO and KEGG pathway thresholds were specified. Additionally, the top 5 notably distinct paths were selected for visual display.

### 4.5 Single Factor Logistic Regression Analysis

Based on the significantly screened gene set mentioned above, a single‐factor logistic regression analysis was conducted using the glm function to group AD and healthy individuals with the threshold *p* < 0.01. The relevant candidate genes were screened and arranged to be included in subsequent research.

### 4.6 Lasso Prognostic Model Construction and Validation

The study used the R language's cv. glmnet (v1.18.0, http://expasy.org/tools/pROC) function to generate the lambda values for the regression model using a 20‐fold Lasso regression. Given the two categorisation criteria of case‐control studies, the study employed the R language's glmnet function to specify family = “binary” for fitting. Following feature screening, genes having correlation coefficients are selected for subsequent analysis. Subsequently, the DEGs with expression differences in the same direction as the training set were then selected and included in subsequent investigations (*p* < 0.05) based on the validation set GSE63061. Following validation, the R language pROC (v1.18.0, http://expasy.org/tools/pROC/) was used to identify the differentially expressed feature genes, and AUC for a single gene was computed in the training and validation sets using the ROC function that comes with the package. The final validated genes were included as key genes in subsequent studies.

### 4.7 PPI Construction of Key Genes

To explore the biological functions of diagnostic model genes, the GeneMANIA database was used (https://genemania.org). PPI analysis was performed on the top 20 interacting genes of diagnostic genes to predict colocalisation, shared protein domains, co‐expression, and correlations between prediction and pathways, as well as provide the biological processes in which diagnostic genes and interacting genes are located.

### 4.8 Prediction of Upstream miRNAs of Key Genes

To explore the RNA regulatory system of key genes in diseases, the study first obtained a list of AD‐related miRNAs, including 125 miRNAs, based on the HMDD database. Secondly, miRNAs (3′UTR, Score > 0.95) interacting with key genes were predicted based on the miRWalk database, and then the intersection of multiple gene miRNAs was extracted using the VENNY tool. Finally, the miRNAs that intersect with key genes in AD are simultaneously incorporated into the miRNA target network.

### 4.9 Transcription Factor Prediction Analysis

Based on the transcription factor prediction provided by iRegulon in Cytoscape, the enrichment score threshold was set to 3.0/2.0 ROC threshold for AUC calculation = 0.03, Rank threshold = 5000, No significant interaction of TF factors between the two genes was found in the predicted results.

### 4.10 Prediction of Small Molecule Drugs and Molecular Docking Analysis

To explore the correlation between diagnostic model genes and small molecules in drug chemistry, the study utilised model genes through Signature Search (https://maayanlab.cloud/sigcom‐lincs/#/SignatureSearch) to predict single gene drug small molecule compounds. The LINCS L1000 Chemical Perturbations (2021) was chosen for drug small molecule prediction results, and the drug molecules with |Z Score| > 1 and *p* < 0.1 was selected for subsequent studies. The next step was to use molecular docking to investigate the binding capacity of putative small molecule compounds with HIBCH. The active components' (ligands') 3D structures were retrieved in SDF format from the PubChem database. The HIBCH receptors were chosen based on the PDB files obtained from the Protein Data Bank (https://www.rcsb.org/). The ligand and protein files were then submitted to the CB‐Dock2 platform for preliminary molecular docking (https://cadd.labshare.cn/cb‐dock2/).

### 4.11 Prediction of miRNAs Which Targeting MGME1

We utilised the target gene prediction database, Mirwalk 3.0 (http://mirwalk.umm.unih‐eidelberg.de/), mirsystem (http://mirsystem.cgm.ntu.edu.tw/index.php), targetscan human 7.2 (http://www.targetscan.org/vert/), mirdb (http://mirdb.org/) to predict miRNA of the target gene of MGME1, the final target genes of were chosen among the intersection genes supported at least by 2 databases. MiRNA mainly acts on the 3′UTR of the target gene, and the 3′UTR region of the target gene can be constructed behind the reporter gene luciferase in the vector. By comparing the changes in reporter gene expression (monitoring the activity of luciferase) after overexpression or interference with miRNA, the inhibitory effect of miRNA on the target gene can be quantitatively reflected.

### 4.12 Detection of Target Genes MGME1 Regulated by miRNAs Using Luciferase Activity

Based on the outcomes of bioinformatical prediction, two potential miRNAs, mir‐922 and mir‐98, may target MGME1. Double luciferase studies were conducted using MGME1 wild‐type and mutant plasmids to investigate whether mir‐922 and mir‐98 was shown to regulate MGME1 directly. Trypsin was used to prepare, break down, and gather the 293T cells. A cell suspension was then created and placed to 24 well plates. There were 5 × 10^4^ cells in each well. The cells were transfected when the density reached 90%–95%. The experiment was divided into six groups: MGME1 3′‐UTR‐NC + mir‐NC group, MGME1 3′‐UTR‐NC + mir‐922/mir‐98 group, MGME1 3′‐UTR‐WT + mir‐NC group, MGME1 3′‐UTR‐WT + mir‐922/mir‐98 group, MGME1 3 ‐' UTR‐MUT + mir‐NC group and MGME1 3′‐UTR‐MUT + mir‐922/mir‐98 group. The amount of plasmid was 0.5 μg/well, and the amount of liposome was 1.5 μL/well. In each group, 3 multiple holes were set, and the experiment was repeated 3 times.

After a 48‐h transfection period, the old medium was disposed, and an adequate amount of PBS (phosphate‐buffered saline) was used to wash the cells. Each well plate contained a volume of 300 μL of Passive Lysis Buffer 1 ×, which was then allowed to react in a refrigerator at 4°C for 20 min, or until the cells were completely lysed. After the aforementioned procedure, the samples were uniformly mixed, shaken for three to 5 minutes (not too vigorously), mixed evenly, and immediately tested. According to the instructions of the Dual‐Luciferase Reporter Assay System, 40 μL of cell lysis buffer was sucked into a Lockwell maxiosorp detection plate, and 20 μL of Luciferase Assay Reagent was added to continue to shake and mix well. Finally, a multi‐functional microplate reader (Tecan Infinite, California, M2009PR) was used to detect the fluorescence value of the firefly fluorescence enzyme within 5 min. Subsequently, a volume of 20 μL of Stop & Glo Reagent was added into each hole to continue to shake and mix the reaction samples well. Ultimately, the reaction samples were kept at room temperature for 3 min, and then an ELISA reader was used to detect the fluorescence value of Renilla luminescence. The fluorescence value f/R of the firefly fluorescence enzyme is the relative activity of firefly luciferase in each group.

## Author Contributions

**Fei Feng:** conceptualization, data curation. **Shoupin Xie:** investigation, methodology. **Yanping Ma:** investigation, resources. **Yafeng Wang:** software, validation. **Fan Zhang**: methodology, supervision. **Hongyan Wu:** project administration, supervision. **Shenghui Huang**: funding acquisition, project administration, writing – original draft.

## Ethics Statement

The authors have nothing to report.

## Consent

All authors agreed to publication of this paper.

## Conflicts of Interest

The authors declare no conflicts of interest.

## Data Availability

All data are available when requested by readers.
